# KCNT1 Channel Blockers: A Medicinal Chemistry Perspective

**DOI:** 10.3390/molecules29122940

**Published:** 2024-06-20

**Authors:** Francesca Di Matteo, Francesca Mancuso, Rita Turcio, Tania Ciaglia, Claudio Stagno, Carla Di Chio, Pietro Campiglia, Alessia Bertamino, Salvatore Vincenzo Giofrè, Carmine Ostacolo, Nunzio Iraci

**Affiliations:** 1 Department of Pharmacy, University of Salerno, Via G. Paolo II, 84084 Fisciano, Italyrturcio@unisa.it (R.T.); tciaglia@unisa.it (T.C.);; 2 Department of Chemical, Biological, Pharmaceutical and Environmental Sciences (CHIBIOFARAM), University of Messina, Viale F. Stagno d’Alcontres 31, 98166 Messina, Italy

**Keywords:** KCNT1 potassium channel, epileptic encephalopathies, medicinal chemistry campaigns, KCNT1 blockers’ identification

## Abstract

Potassium channels have recently emerged as suitable target for the treatment of epileptic diseases. Among potassium channels, KCNT1 channels are the most widely characterized as responsible for several epileptic and developmental encephalopathies. Nevertheless, the medicinal chemistry of KCNT1 blockers is underdeveloped so far. In the present review, we describe and analyse the papers addressing the issue of KCNT1 blockers’ development and identification, also evidencing the pros and the cons of the scientific approaches therein described. After a short introduction describing the epileptic diseases and the structure–function of potassium channels, we provide an extensive overview of the chemotypes described so far as KCNT1 blockers, and the scientific approaches used for their identification.

## 1. Introduction

Epilepsy is one of the most common chronic noncommunicable neurological disorders affecting over 50 million people worldwide [1]. From the clinical point of view, epilepsy is characterized by recurrent brief episodes of involuntary movement (seizures), which can be partial, if limited to specific parts of the body, or generalized, if the entire body is involved. Epileptic seizures are often flanked by loss of consciousness and faecal and/or urinary incontinence [2]. Moreover, together with the predisposition to the epileptic seizures, the cognitive, neurobiological, psychological, and social consequences fall within the definition of epilepsy [2,3]. The aetiology of epilepsy may deeply vary. The epileptic seizures, for instance, may be due to well-known or presumed genetic defects, despite that the genetic aetiology does not exclude the potential involvement of environmental factors [4].

At the same time, epileptic syndromes can be the result of brain injuries (for example, stroke, trauma, and infections) or metabolic alterations [4]. Finally, there are several epileptic diseases that fall within the “unknown cause”, meaning that the nature of the underlying cause is basically unknown [4].

In recent years, several medicinal chemistry campaigns have been carried out in the search for new anticonvulsant drugs (AEDs) with different mechanisms of action, but still, the major problems related to epilepsies concern the lack of an effective pharmacological treatment.

For instance, about one-third of epilepsies are totally refractory to the current medical treatments and a really high proportion of intractable epilepsies during childhood have a significant detrimental impact on the life of patients, including high risk of cognitive and behavioural/psychiatric comorbidity and early mortality [5,6]. When the clinical conditions are characterized by early seizure onset in childhood, electroencephalogram (EEG) abnormalities, and poor prognosis for seizures, they are, usually, referred to as epileptic encephalopathies (EEs) [7].

**Figure 1:**
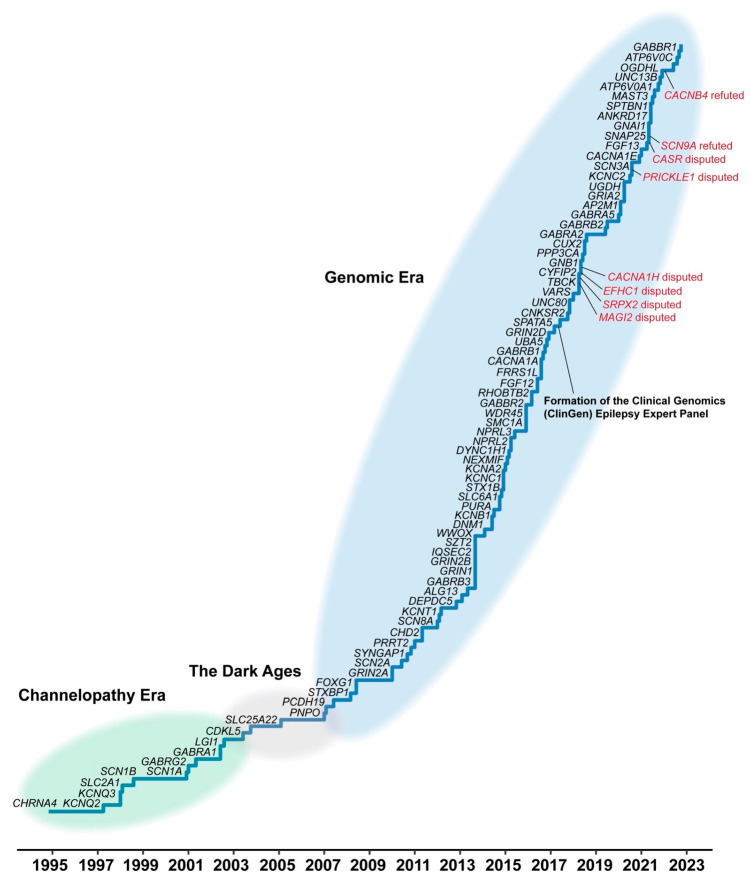
The timeline of gene discovery in the epilepsies (adapted from [17]).

More frequently, patients may have both developmental plateauing and/or regression associated with severe epileptic seizures. This is why, in 2017, the underlying diseases have been more properly classified as developmental and epileptic encephalopathies (DEEs) [8]. DEEs often show a genetic aetiology, and plenty of genes underlying inherited familial epilepsies have been discovered. As shown in Figure 1, the initial identification of genetic disorders leading to DEEs was fast and productive, leading to the discovery of several genes involved in epilepsy, such as CHRNH4 [9], SCN1A [10], SCN1B [11], KCNQ2 [12,13], and GABRG2 [14]. This period is also referred to as the channelopathy era (Figure 1), since the identified genes were mostly encoding ion channels and gave birth to the idea that the genetic epilepsies could be merely considered as a family of channelopathies [15,16]. The enthusiastic beginning of the epilepsy gene discovery era was followed by a period of stagnation where very few novel genes were identified and investigated (the dark era, Figure 1). Then, the development of Next-Generation Sequencing (NGS) and/or Whole Exome Sequencing (WES) techniques strongly boosted the identification of novel genes responsible for DEEs also contributing to demonstrate the significant genetic heterogeneity of these diseases (genomic era, Figure 1).

Since many biological pathways have been implicated in DEEs aetiology, the complete identification of epilepsy with channelopathies has been nowadays strongly questioned. Nevertheless, ion channel misfunction still accounts for a high proportion of genetic epilepsies. It has been reported that mutations to ligand- and voltage-gated ion channel genes account for about 10% and 17% of known epilepsies, respectively [18]. As such, there are many reports unravelling the molecular and neuronal pathways underlying genetic epilepsy caused by mutations in ion channels. Moreover, this is paving the way for the development of disease-targeted therapies in the genetic epilepsies [18].

Among the ion channels involved in DEEs, potassium ones are probably the most promising targets.

## 2. Potassium Channels: General Aspects and Involvement in Epilepsy

**Figure 2:**
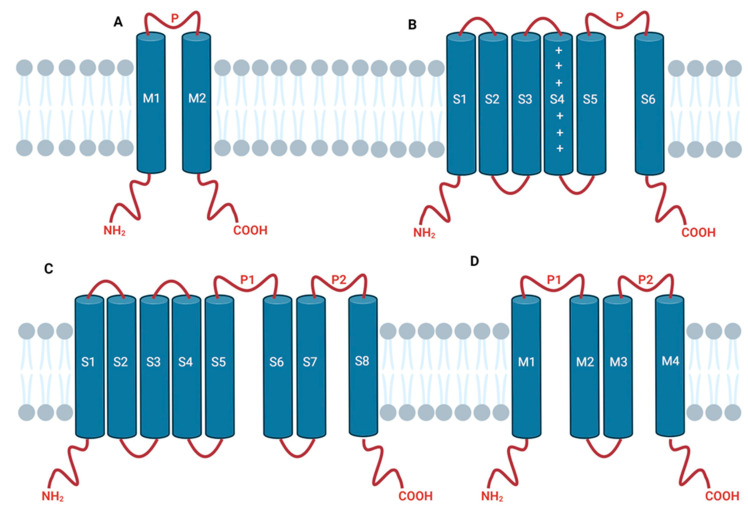
General architecture of potassium channels. (**A**) Inwardly rectifying two transmembrane helices channel 2TM/P. (**B**) Voltage-gated and ligand-gated six transmembrane helices channel (6TM/P, plus signs on S4 represent its role in voltage sensing). (**C**) Eight transmembrane helices/two-pore hybrid channel (8TM/2P). (**D**) Tandem pore domain channel (4TM/2P). Created with BioRender.com, accessed on 4 May 2024.

Potassium channels (KCNs) are the broadest and most heterogeneous class of ion channels, they are encoded by more than 90 genes present in the human genome [19], and, virtually, they are present in every species, both eukaryotes and prokaryotes, except for some parasites [20]. The activity of these channels allows potassium ion flux through the cell membrane, thus generating electric signals in cells. Genetic alterations in these ion channels can generate multiple effects such as the enhancement of neurotransmitters release, altered neuronal excitability, and an abnormal rapid neuronal firing rate [21]. All these effects can be considered clinically relevant in characterizing the epileptic phenotypes, making these ion channels a potential therapeutic target for epilepsy. All KCN channels contain at least a pore-forming domain (PD), composed of two transmembrane (TM) helices that cross the lipid bilayer and are linked by a short loop of about 30 amino acids, usually referred to as the P loop. The amino- and carboxylic-termini are located intracellularly [22,23]. Considering the number of TM helices and the functions, potassium channels could be divided into four main classes (Figure 2) [22,23]:

- Inwardly rectifying channels (2TM/P);
- Voltage-gated and ligand-gated channels (6TM/P);
- Hybrid channels made from the two previously mentioned classes (8TM/2P);
- Tandem pore domain channels (4TM/2P).

The prototypical KCN monomer consists of a PD made of two transmembrane helices linked by a P loop (2TM/P, Figure 2A). This structural motif is almost universal for KCN, but each subfamily of channels is characterized by further, distinct features. The ligand-gated and voltage-gated KCN, for instance, bears four transmembrane helices (S1–S4) preceding the 2TM/P motif (Figure 2B), thus implementing the capability to sense and react to the modification of transmembrane potential. Finally, 4TMs and 8TMs channels can be considered as dimers of two 2TM/P motifs (Figure 2C) and of one 2TM/P motif and one 6TM/P motif (Figure 2D), respectively.

**Figure 3:**
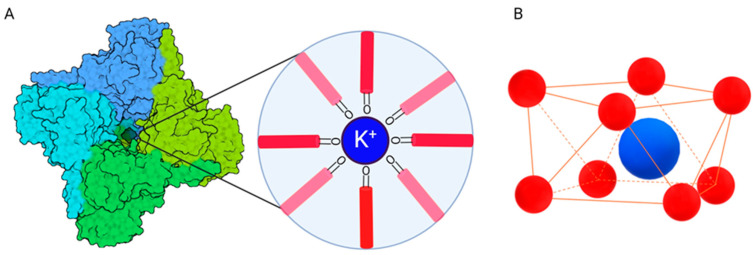
(**A**) Above view of the canonical tetrameric structure of KCNs (each subunit is differently coloured) with magnification of the oxygen checkpoint. (**B**) Typical square-antiprism geometry of a single oxygen checkpoint with the eight oxygens (red) coordinating the potassium ion (blue). Created with BioRender.com, accessed on 4 May 2024.

The canonical structure of KCN consists of a tetramer made by four subunits (Figure 3), which form the pore through which the ions pass; two layers of aromatic amino acids are extended into the lipid bilayer, near the membrane–water interface. The pore region is composed as follows: each subunit directs one PD helix (namely the inner helix) toward the centre of the pore, whilst the second one (namely the outer helix) faces the lipid membrane. The inner helices are angled with the membrane of about 25° [23]. The P loop is constituted by an amino acid sequence of Thr-Val-Gly-Tyr-Gly, whose carbonyl oxygens are an essential part of the so-called selectivity filter (Figure 3). This filter consists of five stereochemical checkpoints, made up by the oxygens of the previously mentioned amino acids. These checkpoint lines are repeated every ~3.0 Å along the filter [24]. Four checkpoints assume a square-antiprism geometry in which the potassium ion is coordinated by eight oxygen atoms that drive the ion along the filter. The selectivity filter is completed by two additional binding sites, one at the external entrance to the filter consisting of four carbonyl oxygens from the filter and four water oxygens, and the second at the internal entrance to the filter, where oxygens are entirely provided by water molecules [25].

6TM/P channels, also known as voltage-gated and ligand-operated potassium channels, are, probably, the most heterogeneous family of KCN channels, and are also the most involved in the aetiology of epilepsy [26]. Among them, Kv7 and KCNT1 channels have progressively gained importance as molecular targets in the treatment of channel-associated DEEs, also considering the high potential for the development of personalized therapies [27,28,29].

The Kv7 (KCNQ) subfamily of K^+^ channels comprises five members (Kv7.1–7.5), each showing distinct tissue distribution and physiological roles. The 7.2 and 7.3 subunits of Kv7 channels are the most abundant in both the central and the peripheral nervous system. They usually assemble as heteromeric channels composed of Kv7.2 and Kv7.3 subunits and are thought to underlie a K^+^ current, named M-current (I_KM_), accounted for the reduction in neuronal excitability and repetitive firing, thus causing spike frequency adaptation. Mutations in the genes encoding for Kv7.2 and Kv7.3 (i.e., KCNQ2 and KCNQ3, respectively), mainly leading to loss-of-function (LoF) phenotypes, are responsible for a wide range of epileptic diseases [12,13,30]. This is the reason why Kv7.2/7.3 openers are widely investigated as potent anticonvulsants in humans, with a couple of candidates that have reached clinical trials [31,32,33,34,35,36,37].

On the other hand, despite belonging to the 6TM family, KCNT channels are sodium-activated potassium channel (K_Na_), generating the so-called IK_Na_ current, initially discovered in mammalian cardiac cells [38] and, lately, also in avian trigeminal ganglion neurons [39]. The current is generated in reply to the increase in the intracellular concentration of sodium ions as a consequence of repeated action potentials, leading to KCNT channels opening [40]. KCNT channels are known to be encoded by two genes belonging to the Slo family (Slo 2.2 and Slo 2.1), which are also known as the Slack (KCNT1) and Slick (KCNT2) channels, respectively. [41] Similarly to the other TM6 potassium channels, KCNT channels are assembled as tetramers in which each subunit is composed of a transmembrane domain (six helices, S1–S6) with S1–S4 forming the voltage sensor domain (VSD) and S5–S6 constituting the pore-forming domain (PD). A large cytoplasmic domain is composed by two “regulator of K^+^ conductance” (RCK) domains and a highly conserved PDZ-binding domain at the C-termini [41,42].

KCNT channels are expressed in many different types of neuronal cells and several locations [43,44] rather in neurons and glia cells [45].

Despite mutations in KCNT2 having been recently reported also to cause DEEs [46,47,48], it is widely known that mutations in KCNT1, which are mostly responsible for Gain-of-Function (GoF) phenotypes, account for severe, drug-resistant rare forms of childhood epilepsy, including Epilepsy of Infancy with Migrating Focal Seizures (EIMFS) [49], Autosomal Dominant Sleep-Related Hypermotor (Hyperkinetic) Epilepsy (ADSHE) [50,51], Ohtahara [52], West [53], and Lennox–Gastaut syndromes [54]. KCNT1-related encephalopathies are also characterized by developmental regression and/or intellectual disabilities [54].

At the molecular level, different mechanisms underlying the enhanced channel functioning of GoF KCNT-1 variants have been proposed, among which are the following: the suppression of subconductance states together with changes in protein kinase C regulation [49]; changes in Na^+^ sensitivity [55]; increased channel cooperative gating together with decreased single-channel conductance [56]; and altered interactions with binding partners [57,58]. Considering the GoF variants as the most pathogenic KCNT1 variants, during recent years, several efforts have been made by both academia and industry to discover KCNT1 blockers suitable as therapeutic tools for precision medicine.

## 3. Medicinal Chemistry Strategies for Identification of KCNT1 Blockers

### 3.1. Drug Repurposing

**Figure 4:**
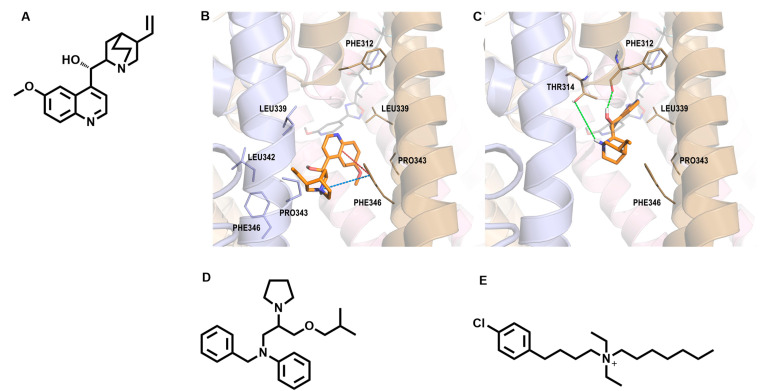
(**A**) Molecular structure of quinidine. (**B**,**C**) In silico predicted binding poses of QND into KCNT1. In both panels, the QND bound conformation is represented as orange sticks. KCNT1 monomers are depicted as light blue, sand, light pink, and wheat cartoons, whereas residues interacting with the ligand are represented as solid sticks. Cation-π and π-π stacking interactions are highlighted by blue and red dashed lines, respectively. H-bonds are represented by green dashed lines [74]. (**D**) Molecular structure of bepridil. (**E**) Molecular structure of clofilium.

Quinidine (QND—Figure 4A), a class IA antiarrhythmic agent, primarily acting by voltage-gated sodium channel blockade, is the first molecule to be identified as a KCNT1 blocker by drug repurposing approaches. Its clinical use for the treatment of KCNT1-related epilepsies was initially investigated by in vitro experiments [59] and clinically challenged later [29,60,61]. Despite that initial results were quite encouraging, the extensive use of quinidine in KCNT1-related epilepsies was strongly questioned when larger and systematic clinical studies were performed. These studies highlighted heterogenous anticonvulsant efficacy, ranging from positive responses to a lack of efficacy, often penalized by severe toxicity [62]. Several reasons can be given to rationalize this evidence. Quinidine is a rather weak KCNT1 blocker (~80 < IC_50_ < 100 μM [63,64]), meaning that several factors, including the severity of the disease, the specific molecular defect, the age of EE onset, and quinidine therapy initiation, can deeply affect its efficacy. Moreover, quinidine has an unfavourable pharmacokinetic profile and in spite of its rapid adsorption in the gastrointestinal tract and its rapid tissue distribution, it shows really poor brain penetration and an extensive liver metabolization by the cytochrome-P450 enzyme system [65,66]. The cytochrome P-450-dependent metabolism is also responsible for the high number of drug–drug interactions shown by the molecule, including inhibition of the metabolism of many other antiepileptic medications [60]. Finally, quinidine is characterized by a well-assessed polypharmacology relying on the simultaneous modulation of NaV1.5, Kv1.4, Kv4.2 [67], and hERG channels, not to mention its effects as a muscarinic receptor antagonist, a-1 blocker, and antimalarial agent. In particular, the inhibitory potency of quinidine over hERG, that is 100-fold higher than the potency shown in the inhibition of KCNT1 currents [68,69], accounts for its most insidious and recurrent side effect: the modulation of cardiac conductance by QT prolongation and torsades de pointes [70]. These are the reasons why the clinical use of quinidine in KCNT1-related DEEs is nowadays extremely limited. At the same time, the empirical use of quinidine did not provide much evidence concerning the molecular determinants driving KCNT1 current inhibition. It has been reported that quinidine binds into the KCNT1 intracellular pore vestibule, immediately below the selectivity filter, establishing a pivotal interaction with Phe346 [71,72]. This is the reason why KCNT1 channel mutants, such as F346L and F346I, are almost insensitive to the alkaloid [71,72]. Nevertheless, despite both chicken and human KCNT1 cryo-EM coordinates having been released [42,73], the KCNT1/quinidine complex has never been resolved. Very recently, a molecular dynamics (MD)-based in silico study has postulated the existence of two distinct binding modes that quinidine bounces between [74]. In the first bound conformation, corresponding to the one most populated in the MD trajectory (Figure 4B), quinidine is located between two S6 helices belonging to two adjacent KCNT1 subunits. Within this binding site, quinidine establishes hydrophobic interactions with Leu339, Pro343, and Leu342 residues, also having additional π-π and π-cation interactions with Phe346 [74]. In its second bound conformation (Figure 4C), quinidine is instead flipped upside down and shifted toward the pore helix, interacting via a direct H-bond with the backbone of Phe312 and via a water-mediated H-bond with Thr314. Additional π-π stacking interaction is observed with Phe312 (Figure 4C). [74]

Due to the substantial failure of quinidine-based therapies for KCNT1-related DEEs, additional molecules have been proposed by drug repurposing. For instance, bepridil (Figure 4D), a drug with direct negative chronotropic, dromotropic, inotropic, and vasodilatory actions mediated by the inhibition of calcium channels [75], is also found to be able to inhibit KCNT1-mediated currents [64,76]. The reported bepridil IC_50_ (6.36 ± 2.12 μM) is even lower than that of quinidine, with whom bepridil also putatively shares the KCNT1 binding site [71]. Nevertheless, the safety of the drug is even lower than quinidine and the prolongation of QT intervals and torsade de pointes due to hERG inhibition again represent the most severe drawback [76]. Another repurposed antiarrhythmic drug, clofilium (Figure 4E) [77], has been proposed as a therapeutic tool for KCNT1-related epilepsies [78], besides being devoid of clinical significance. Clofilium blocks KCNT1 with an IC_50_ similar to the quinidine one (~100 μM) but is extremely more potent in the inhibition of hERG channels (IC_50_~2.5 nM) [79] and also shows a molecular structure and a pharmacokinetic profile unfit for a CNS drug.

### 3.2. High-Throughput and Virtual Screening Approaches

**Figure 5:**
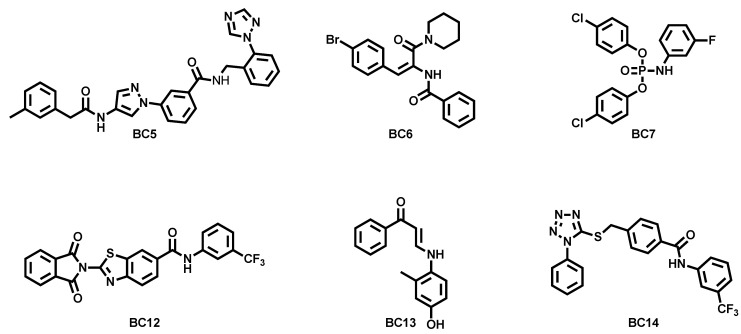
Structures of a first series of KCNT1 inhibitors identified by virtual screening studies [71].

The first description of a medicinal chemistry workflow leading to the identification of KCNT1 blockers was reported by Cole and coworkers [71]. They modelled the quinidine binding site in silico, starting from the above mentioned cryo-EM structure of chicken KCNT1 [42]. The following virtual screening protocol over a diversity-based library of 100,000 drug-like molecules led to the selection of 17 molecules based on their docking score and commercial availability. The selected molecules were challenged for their ability to block KCNT voltage ramp-evoked currents in patch-clamp experiments. These assays led to the identification of six KCNT1 inhibitors (BC5–BC7 and BC12–BC14, Figure 5) with greater potencies when compared to both quinidine and bepridil, which were used as reference compounds (0.6 < IC_50_ < 7.41 μM). When tested over the disease-causing GoF mutant Y796H, compounds retained micromolar IC_50_s, despite a significant decrease in potency being evidenced for the most potent blockers (BC7 and BC14) in comparison with the wild-type channel. All the compounds were well tolerated in a preliminary cell-based toxicological assay and, notably, two of them (BC12 and BC13) showed weak inhibition (~10–20%) of hERG channels at 10 μM. Due to the high chemical diversity of the identified molecules, no evident structure–activity relationships related to KCNT1 blocking could be obtained by this report, also considering that no hit-to-lead development has been reported in the literature so far.

**Figure 6:**
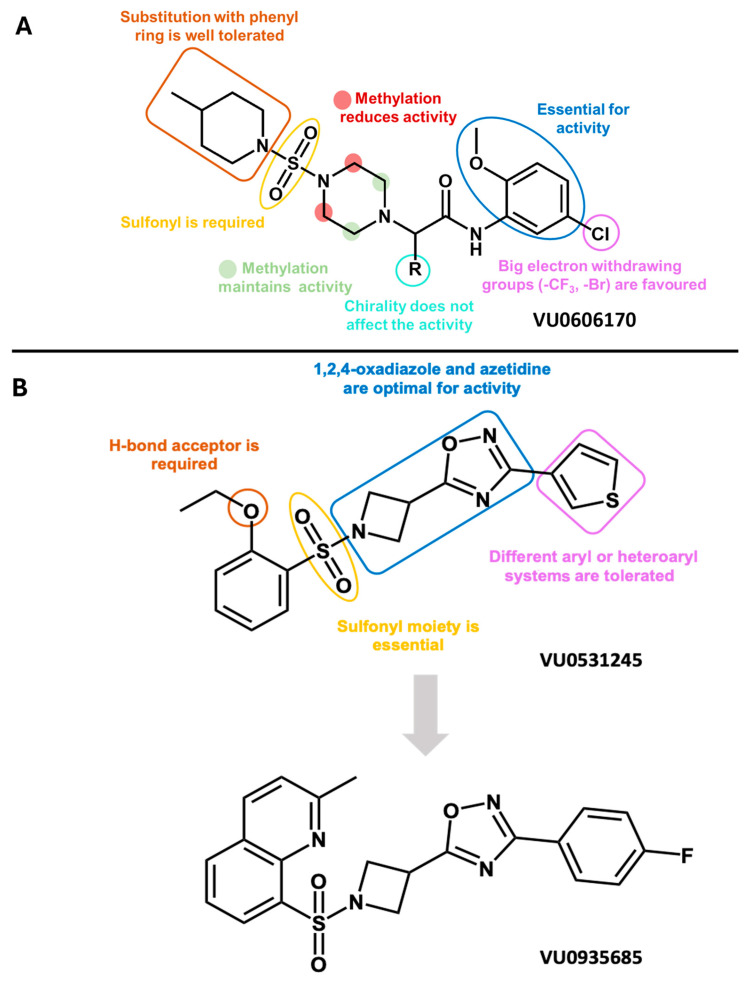
(**A**) Structures of VU hit compounds and (**B**) corresponding SAR maps [79,80,81].

In 2020, Spitznagel and coworkers used a slightly different approach to the identification of KCNT1 modulators: they screened a molecular library of 100,000 compounds using a high-throughput thallium-based (TI^+^) flux assay and HEK293 cells engineered to express both wild-type and the disease-related (GoF) KCNT1 A934T mutant [79]. The screening database was built up starting from different commercially available libraries and the selection was made in order to provide lead-like motifs, minimum pan-assay interference, and maximum diversity. The high-throughput screen (HTS) had a hit identification rate of 0.5%, and the active molecules were grouped by structural features, with the aim of decreasing the time and cost of the screening, although the clustering method has not been described. Only representative compounds belonging to each structural class were then retested (44% of the initially discovered hits). Selectivity assays over HEK293 cells expressing many different ion channels (KCNT2, Slo, GIRK1/2, Kv2.1, TREK1, hERG, NaV1.7, and Cav3.2) were performed, along with the evaluation of the activity of the initial hit selection over the epilepsy-related KCNT1 mutants G288S and R428Q. The results allowed for the identification of the most promising compounds that, in turn, were challenged in dose–response curves against WT and A934T KCNT1, finally culminating with the identification of VU0606170 (Figure 6). In the thallium flux assay, the latter compound showed an IC_50_ of 1.84 μM and 1.16 μM in WT and A934T KCNT1, respectively, and the micromolar potency was confirmed by whole cell automated patch-clamp recordings over the same cell cultures (IC_50_s = 2.43 μM and 2.06 μM over WT and A934T KCNT1, respectively). The molecule showed no activity at a 10 μM concentration over the KCNT2, Slo, GIRK1/2, Kv2.1, TREK1, NaV1.7, and Cav3.2 ion channels, while it inhibited hERG channels by 36% [79]. Notably, when administered to a spontaneously firing cortical neuronal culture, composed of a mixture of inhibitory, excitatory neurons and astrocytes, VU0606170 produced a decrease in the spiking rate of 55.58% with an IC_50_ of 3.70 μM, also inducing a dose−response decrease in frequency of intracellular Ca^2+^ baseline oscillations. The same research group attempted the optimization of VU0606170 by systematic chemical replacement of the piperidinyl and phenyl substituents, of the sulfonyl and acetamide linkers, and of the piperazine central core (Figure 6A) [80]. The obtained results were not particularly encouraging since no VU0606170 derivatives showed any substantial improvement in potency. The phenyl ring seems essential for the activity of this series of compounds, as well as the alkoxy substituent in position 2. Any replacement of those groups is responsible for a complete loss of activity or a shift from inhibition to activation. The substituent in position 5 of the phenyl ring also plays a pivotal role: larger electron withdrawing groups (such as trifluoromethyl and bromine) are favoured over smaller and electron-donor groups. Methylation of the acetamide linker mostly retains the KCNT1-blocking activity independently of the chirality of the corresponding derivative. The piperazine ring was also modified by methylation. When methyl was placed close to the sulphonamide moiety, activity was reduced, while it was maintained when the methyl group was positioned close to the methyl acetamide. Any attempt to replace the piperazine ring with structural analogues failed to provide activity as well as the replacement of the sulfonyl linker with a carbonyl moiety. Finally, the replacement of 4-methylpiperidine always provided a loss of activity or a shift to KCNT1 activation, except for the use of a phenyl ring, which did not significantly alter the KCNT1 inhibition potency. Despite providing some interesting results, this report also does not provide evidence in terms of structural determinants for KCNT1 inhibition: the resulting SAR was relatively flat and, likely, limited to VU0606170 analogues, without providing clear and solid clues for rational design and hit-to-lead development. This could be due, in the opinion of the authors, to the ability of the tested analogues to modulate KCNT1 function by a mechanism other than pore blockade and is also probably related to the binding site occupied by this class of molecules, tolerating only minimal structural modifications. Nevertheless, a rational interpretation of the results by the use, for instance, of molecular modelling or cryo-EM studies might make these data more exploitable.

The same research group later reported KCNT1 blockers based on a 1,2,4-oxadiazole scaffold (Figure 6B) [81]. This scaffold was probably selected by cross-checking their previous results with the patent literature data. Indeed, Praxis Precision Medicine patented several compounds, characterized by the presence of a common 1,2,4-oxadiazolyl moiety, as KCNT1 blockers [82,83]. The authors realized that one of the hit compounds identified in their very first cell-based HTS campaign [79] was actually a 1,2,4-oxadiazole derivate (VU0531245, Figure 6B) that had been initially excluded from further investigations (vide supra). The compound was a relatively potent KCNT1 blocker (IC_50_ = 2.1 μM) and showed high cell permeability and good calculated properties as a CNS permeant [81]. Nonetheless, the hit compound showed a serious pharmacokinetic drawback: an extremely high metabolic clearance when tested, in vitro, using the mouse liver microsomes metabolism model [81]. This is why the researchers attempted a scaffold optimization, taking into account both pharmacokinetic and pharmacodynamic features. Analogously to their previous paper, in this one, they performed a systematic scanning of the different molecular moieties of VU0531245, reporting interesting results that unfortunately are somewhat difficult to be rationalized. Modification of the phenyl ring by using different electron-withdrawing and electron-donating groups, as well as different alkoxy, pyrrolidinyl, and piperazinyl substituents, was mostly detrimental, leading to a loss of activity or mode switching from an inhibitor into an activator. Only a few compounds maintained inhibitory properties comparable to the parent molecule VU0531245. Taken together, these results led the author to hypothesize about the critical role of a hydrogen-bond acceptor at position 2 of the phenyl ring for KCNT1 inhibition. In fact, when this pharmacophoric chemical feature was maintained, it was possible to replace the phenyl ring with more complex bicyclic analogues, that mostly maintained or even improved the KCNT1 inhibitory potency [81]. Modification of the VU0531245 in any other point gave inactive or faintly active derivatives. For example, any attempt to replace the sulphonamide linker by different moieties, such as methylene, ethylene, amide, urea, and carbamate, did not improve the compound activity. The authors rationalized these results based on the peculiar sulphonamide geometry, that presumably provides the correct mutual orientation of the different functional groups. Similarly, the replacement or modification of the 1,2,4-oxadiazole or azetidine ring was not fruitful, while only minimal modification of the thiophenyl moiety was tolerated. In particular, the authors have proposed the introduction of halogen atoms in position 2 and 4 of the thiophenyl ring that, besides only grossly maintaining the inhibitory potency, are useful to block a metabolic hot spot of the parent molecule [81]. Collectively, ten molecules were selected for further investigation by patch-clamp experiments. They all featured low micromolar potencies versus WT and A924T KCNT1, as well as some selectivity. Indeed, when challenged over Slo and KCNT2 potassium channels at the concentration of 10 μM, almost all the compounds were inactive. The most potent of the series, compound VU0935685 (Figure 6B) was also reported as inactive on hERG at the same concentration. Since no clear pharmacodynamic improvements were attained, the authors also pharmacokinetically challenged their molecules by the assessment of metabolic stability in mouse liver microsomes (MLMs) and by plasma protein binding testing. Unfortunately, the results were mostly unsatisfactory: the analogues suffered from the same high metabolic instability that characterized the parent molecule [81]. As in the previous reports by this research group, results were not rationalized by the use of molecular modelling techniques.

To date, the biopharmaceutical company Praxis Precision Medicine is the only one that has disclosed an advanced discovery project for the development of small-molecule therapeutics for the treatments of KCNT1-related epilepsies [82] These efforts led to the identification of the first orally available KCNT1 inhibitor (**17**—vide infra) described so far, identified through extensive SAR studies enclosed in several patent applications [82,83,84].

**Figure 7:**
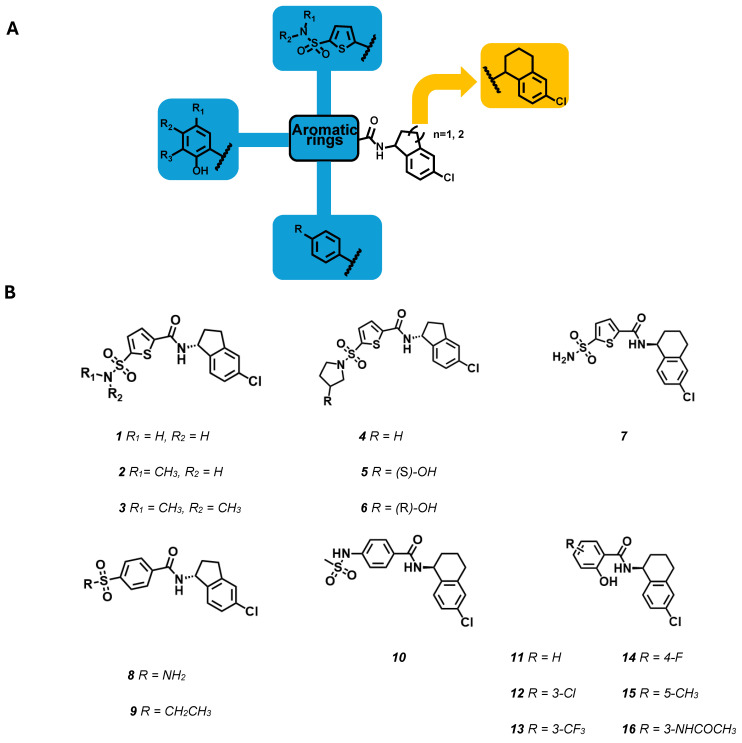
(**A**) SAR map for the 5-chlorindanyl and 6-chlorotetraline derivatives. (**B**) Structure of KCNT1 inhibitors patented by Praxis Precision Medicine [82,83] (**1**–**16**).

The compounds reported in the first patent application share common structural features: the 5-chloroindanyl or 6-chlorotetraline moieties are critical elements for this series of compounds (**1**–**16**). This basic moiety is connected via an amide linker to different aromatic systems, thus generating three distinct subseries containing a thiophenyl, phenyl, or phenol ring (Figure 7A).

Looking at the thiophenyl series, the authors observed that the sulphonamide moiety could be functionalized with aliphatic chains (**2**–**3**) or incorporated in alicyclic rings (e.g., pyrrolidine ring, **4**–**6**) resulting, in some cases, in potent compounds. Attending to phenyl and phenolic subseries, the following substitution patterns were observed: in the phenyl case, the *para* substitution is preferred (**8**–**10**), whilst in the other one, the introduction of both electron-donor or electron-withdrawing groups in the positions 3, 4, and 5 led to high inhibiting potency (**11**–**16**). The role of the sulphonamide group has been investigated also among the phenyl derivatives, where it was shown again that the introduction of alkyl chains led to an enhancement in potency if compared to the unsubstituted one (**8**). However, the presence of sulphonamide is not crucial for the activity as demonstrated by the active compounds bearing sulphone (**9**) or inverted sulphonamide (**10**) moieties.

**Figure 8:**
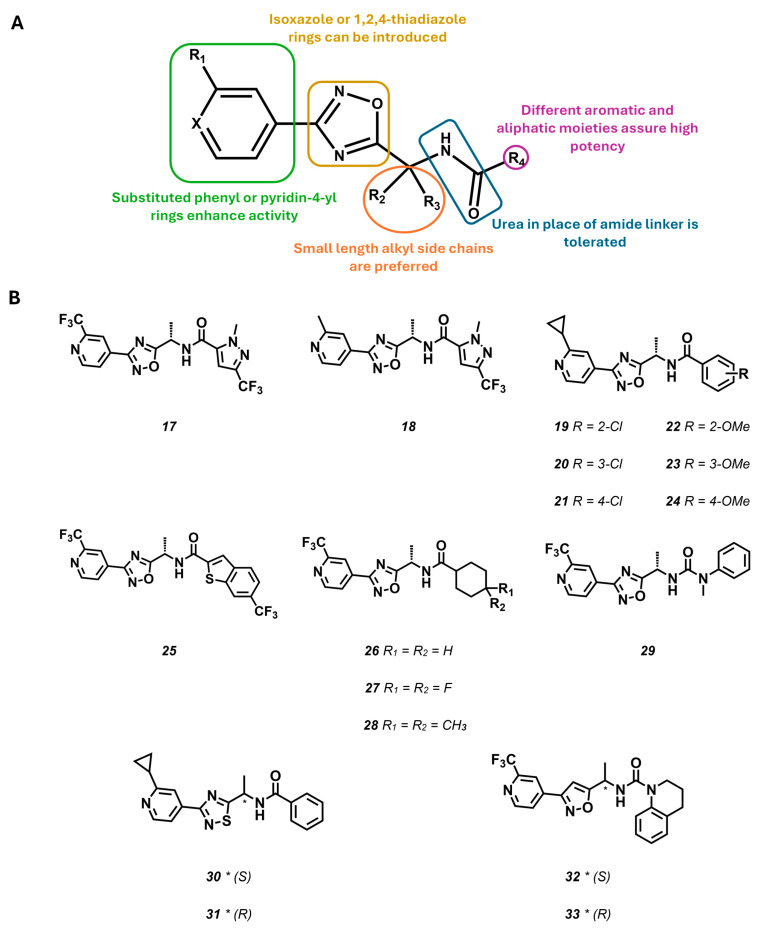
(**A**) SAR map of the 1,2,4-oxadiazole, 1,2,4 thiadiazole, and isoxazole derivates. (**B**) Structure of KCNT1 inhibitors patented by Praxis Precision Medicine (ref [82] and references therein) (**17**–**33**). * indicates a chiral center.

The second and third patent applications present the results of an extensive pharmacological study for 1,2,4-oxadiazole derivatives [82]. A general chemical structure for this class of compounds is depicted in Figure 8. The introduction of a substituted or unsubstituted phenyl or 4-pyridinyl ring on the 1,2,4-oxadiazole core led to compounds characterized by high potency. Substituents introduced on this aromatic core exert a wide influence on the compounds pharmacological profiles: substitution at the 3-position was carried out with methyl (**17**–**18**), trifluoromethyl (**25**–**29**), or cyclopropyl (**19**–**24**) groups, often leading to potent compounds. Further SAR analysis highlighted the role of the methylene linker, where the introduction of the methyl side chain is generally associated with an improvement in the inhibitory activity. The role of the stereochemistry is barely inferable since only in a few cases was the (*S*)-enantiomer found to be the eutomer, while in the other ones, both stereoisomers showed the same activity profile.

Considering the results of the first SAR investigation round, and the high lipophilicity of the synthesized derivatives, the authors investigated whether polar heterocycles at R4 could affect KCNT1 inhibitory potency, while improving pharmacokinetic properties. Concerning this region, the influence of a pyrazole ring with a different substitution pattern was initially investigated. The most interesting results were obtained for the N1–C3 substituted pyrazoles, especially for the combination of methyl and cyclopropyl (**17**) or methyl and trifluoromethyl (**18**) groups. In more detail, it was noted that the relocation of substituents (e.g., methyl) to N2 of the pyrazole ring led to a complete loss of activity. Several other rings have been substituted to pyrazole, highlighting how even extensive structural changes in this region do not compromise activity (**19**–**29**). Indeed, SAR studies indicate that six-membered ring, aromatic (**19**–**24**), or aliphatic (**27**–**28**) as well as fused heterocycles (**25**, **32**–**33**) in place of the pyrazole ring are well tolerated. Further substitution of the amide linker with a urea one (**29**) has also been reported through these patents showing that, even in this case, the bioactivity is retained. More recently, the role of the central 1,2,4-oxadiazole has been significantly explored by preparing 1,3,4-oxadiazole, pyrazole, 1,2,4-thiadiazole, and isoxazole analogues. The replacement of the central core suggested that this portion of the molecule does not simply act as linker, but the electronic profile of this ring contributes to the biological activity. Indeed, the isosteres 1,3,4-oxadiazole or pyrazole failed to act as KCNT1 blockers.

Compound **17** (Figure 8) is a potent KCNT1 inhibitor, with a suitable pharmacokinetic profile and is also the sole molecule suitable for in vivo models, given that all the other congeners were, unexpectedly, dramatically less active over mouse KCNT1 variants [84]. Compound **17**, in fact, showed an IC_50s_ of 40 nM and 622 nM in hKCNT1 and mKCNT1, respectively, also showing remarkable potency for cynomolgus monkey (49 nM) and rat KCNT1 (545 nM), when challenged in patch-clamp experiments. The compound also featured nanomolar or low micromolar potencies over different epilepsy-related hKCNT1 variants (221 nM ≤ IC_50_s ≤ 1768 nM). The compound selectivity was further assessed over a panel of 80 targets at a 10 μM concentration (for the full list of targets, please see the supplementary information file of reference [84]), using binding displacement assays. The compound showed activity over only two targets: TSPO (63% displacement) and GABA_A_ (74% displacement). Patch-clamp measurements using compound **17** over other different ion channels, such as hERG, hNaV1.5, Cav1.2, IKs, BK, and KCNT2, revealed its selectivity (10.7 μM ≤ IC_50_s ≤ 42 μM). As for the previous reports, none of the above-described results were rationalized in terms of target engagement, thus reducing the overall impact of the research. The activity of compound **17** was further challenged in a native mouse tissue brain slice by whole-cell patch-clamp recordings. The effects on neuronal firing were evaluated both in slices from WT mice and from mice homozygous for mKCNT1-P905L (Kcnt1^L/L^), that is known to recapitulate many key features of the EEs, including spontaneous seizures, high interictal spike frequency, and reduced survival [85]. Compound **17** did not alter the normal firing of wild-type mouse slices, that was, instead, significantly decreased in neurons from Kcnt1^L/L^ mice when a 10 mM concentration was reached. Preliminary in vivo pharmacokinetic testing of compound **17** revealed suitable brain penetration and clearance values for in vivo pharmacological testing, after oral dosing [84]. Nevertheless, it is unclear why the molecule was then tested in vivo in the Kcnt1^L/L^ model, using a subcutaneous administration regimen. In this experimental set up, compound **17** at a 30 mg/Kg dosing showed a remarkable improvement in the mouse phenotype with an acceptable therapeutic window [84]. It is worthy to note that, given the effect of the molecule over GABA_A_, further evidence should be provided to exclude the involvement of GABAergic modulation on the observed antiepileptic effects.

**Figure 9:**
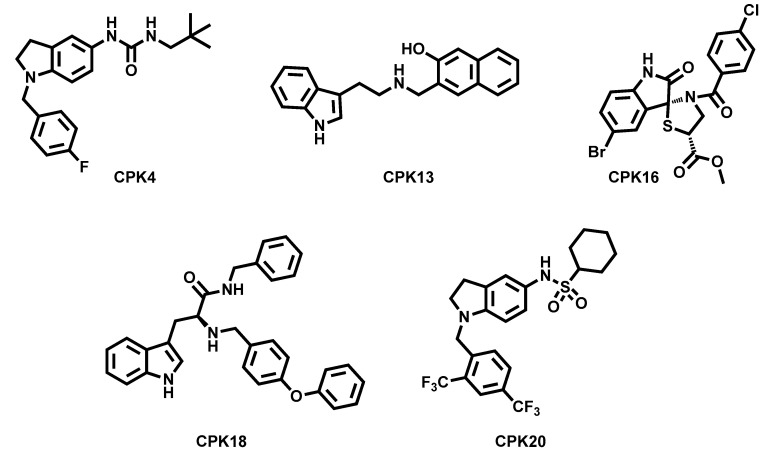
Structures of the CPK hit compounds [74].

Finally, our research group has recently reported the identification of distinct chemotypes as KCNT1 inhibitors, identified through a virtual screening approach [74]. The screening was based on a model of the human KCNT1 modelled by homology with the cryo-EM structure of the chicken channel in open conformation [23], since, when the investigation started, the cryo-EM coordinates of human KCNT1 [73] had not yet been released. Molecular dynamics-based docking of quinidine into the intracellular pore vestibule of the model was performed to search for the most favourable quinidine/KCNT1 complex conformations. The 50 lowest-energy quinidine/target conformations were then used to compose a docking target ensemble that was used to screen in silico an in-house compounds database (~2000 compounds). Among the top scoring compounds, 20 molecules were selected based on their chemical diversity. The selected molecules were challenged as KCNT1 blockers, using a Tl^+^ flux assay in CHO cells stably expressing hKCNT1 channels and using loxapine as a channel opener. A total of 5 out of 20 compounds showed remarkable ability in antagonizing loxapine-evoked KCNT1 currents (CPK compounds, Figure 9). The five compounds showed improved potency (3.4 ± 0.70 μM ≤ IC_50_s ≤ 12.2 ± 2.60 μM) over quinidine (IC_50_ = 147 ± 31 μM), used as a reference compound. Results obtained by fluorimetric assays were confirmed in patch-clamp experiments, in which the selected molecules also proved to have slower blocking kinetics than quinidine with an improved affinity to the channel binding site. In fact, the effect of quinidine was completely reverted upon drug wash-out, while the extent of current recovered upon removal from the perfusion bath of the five compounds was much lower. These results have been rationalized by the mean of molecular modelling studies using the cryo-EM structure of hKCNT1 [73]. Because of the chemical diversity of the identified hits, no precise SAR conclusions were drawn, but, by the use of in silico and in vitro techniques, hypotheses were provided about the inhibitor/channel binding hot spots [74]. All the CPK compounds were unable to modulate Kv7.2 channels, while three of them (CPK16, 18, and 20) did not exert blocking activity over hERG at a 10 μM concentration. Also, these results were rationalized in terms of specific chemical features of the different chemotypes. When challenged in vitro for their metabolic stability, compound CPK20 emerged as the most stable, additionally showing lower potencies in blocking KCNT2 channels, while similar (when compared to WT channels) potencies were observed in blocking pathogenic GoF KCNT1 mutants G288S and A934T.

## 4. Conclusions

In summary, none of the compounds described in this review can provide conclusive proof that KCNT1 inhibition will produce the desired anti-epileptic therapeutic effect in vivo. Moreover, the in vitro results are mostly penalized from the lack of a rational structure–activity relationship investigation that does not allow, at least at the present stage, a rational design of a new series of KCNT1 blockers. Thus, there is a need to continue the discovery efforts directed toward the identification of such compounds, that could be extremely facilitated by the experimental resolution of different KCNT1 inhibitors in complex with the target channel. Moreover, pharmacokinetic refinement of the identified molecules will be necessary, considering that it is mandatory for this class of compounds to have sustained exposure in the CNS. Finally, animal models of KCNT1-related epilepsies are necessary to preclinically validate identified lead compounds and to clearly link their potential anti-epileptic effects to the sole KCNT1 modulation.

## Data Availability

Not applicable.
